# Symmetry of palatal shape during the first year of life in healthy infants

**DOI:** 10.1007/s00784-020-03403-4

**Published:** 2020-06-24

**Authors:** R. Bruggink, F. Baan, G.J.C. Kramer, A.M. Kuijpers-Jagtman, S.J. Bergé, T.J.J. Maal, E.M. Ongkosuwito

**Affiliations:** 1 Department of Dentistry - Orthodontics and Craniofacial Biology, Radboud University Medical Center, Philips van Leydenlaan 25, 6525 EX Nijmegen, The Netherlands; 2 3D Lab Radboudumc, Radboud University Medical Center, Geert Grooteplein Zuid 10, 6525 GA Nijmegen, The Netherlands; 3 Alkmaarse Orthodontisten, Noordwest Ziekenhuisgroep, Wilhelminalaan 12, 1815 JB Alkmaar, The Netherlands; 4 Department of Orthodontics, University Medical Center Groningen, Hanzeplein 1, 9713 GZ Groningen, The Netherlands; 5 Department of Orthodontics and Dentofacial Orthopedics, School of Dental Medicine/Medical Faculty, University of Bern, Hochschulstrasse 4, 3012 Bern, Switzerland; 6 Faculty of Dentistry, Universitas Indonesia, Jakarta, Indonesia; 7 Department of Oral and Maxillofacial Surgery, Radboud University Medical Center, Geert Grooteplein Zuid 10, 6525 GA Nijmegen, The Netherlands; 8 Amalia Cleft And Craniofacial Centre, Radboud University Medical Centre, Geert Grooteplein Zuid 10, 6525 GA Nijmegen, The Netherlands

**Keywords:** Orthodontics, Dental models, Imaging, three-dimensional, Diagnostic imaging, Maxillofacial development

## Abstract

**Objectives:**

The purpose of this study was to quantify the symmetry of the alveolar process of the maxilla and palate during the first year of life in healthy infants with the help of a semiautomatic segmentation technique.

**Materials and methods:**

Maxillary plaster models of seventy healthy babies at 0, 3, 6, 9, and 12 months were collected and digitized. A semiautomatic segmentation tool was used to extract the alveolus and palate. The resulting model was aligned within a reference frame and mirrored on its medial plane. Distance maps were created and analyzed to compare and quantify the differences between the two hemispheres. Additional hemispherical width and area measurements were performed. An ANOVA test with additional post hoc tests was performed to check if the symmetry changed during development. Finally, the results were tested on intra- and interobserver variability.

**Results:**

The absolute mean inter-surface distance between the original and mirrored models in each age group ranged between 0.23 and 0.30 mm. Width and area analysis showed a small but significant larger left palatal hemisphere. ANOVA and post hoc tests showed no significant difference in symmetry between groups. Reliability analysis showed no significant differences between observers.

**Conclusions:**

This study showed that in this infant population, only a small degree of palatal asymmetry was present, which can be considered as normal and clinically irrelevant.

**Clinical relevance:**

The data from this study can be used in future comparative studies as reference data. Furthermore, modeling of these data can help in predicting the growth pattern, which may lead to improved treatment protocols for children with craniofacial anomalies.

**Electronic supplementary material:**

The online version of this article (10.1007/s00784-020-03403-4) contains supplementary material, which is available to authorized users.

## Introduction

The human body tends to be symmetrical with respect to its vertical midline. Perfect symmetry is extremely rare in living organisms due to genetic imperfections, environmental factors, and functional deviations. In the clinically setting, Fischer et al. [1] described symmetry as balance while significant asymmetry as imbalance of the structure. Larger asymmetries can be found in congenital disorders like orofacial clefts and hemifacial microsomia [2].

In dental development, the symmetry of both the maxillary and mandibular arch is needed for a normal transversal occlusion [3]. During embryological development, genetic defects can have influence on the development of the first branchial arch, which can result in defects like micrognathia, facial asymmetry, clefts, and malocclusions [4]. The symmetry can also be affected due to environmental effects like trauma, infection, and unbalanced skeletal muscular activity [2, 5]. Some studies even conclude that intubation in the neonatal phase could cause palatal asymmetry during development. For example, a study of Kopra et al. [6] showed significant asymmetry in the posterior part of the palate in children between 3 and 5 year old who were intubated as neonates. However, these studies are not all unanimous [6, 7]. To regain normal occlusion, orthodontic treatment and in some cases rapid maxillary expansion or surgical treatment is needed [2].

As some degree of asymmetry is present in the healthy population, the normal amount of asymmetry must be investigated to create a range in which deviations can be considered as normal or divergent. Multiple studies have investigated arch or palatal symmetry. Al-Zubair [8] investigated dental arch symmetry in healthy adults between 18 and 25 years. In this study, linear measurements were performed on adult dental models and showed that there were no significant differences between both hemispheres of the maxillary arch. A study of Moreira et al. [5] analyzed the symmetry of dry skulls derived from subjects aged from 7 months to over 55 years. They showed that some degree of hard palate asymmetry was present, especially in the posterior part of the palate. However, none of these hemispherical deviations were significantly different. Due to the fact that these measurements were performed on dry skulls, it was impossible to monitor the asymmetry of a single person over time. To our knowledge, only little research has been performed about the base of the alveolar arch, in other words the palate and the alveolar process. Data of alveolar arches in healthy infants are even more scarce. Availability of these data, and more specific changes of alveolar arches over time, can provide valuable information to optimize current treatment strategies in children with craniofacial asymmetries (e.g., patients with orofacial clefts, craniofacial microsomia, and congenital malformations). Additionally, such data could be used in other comparative studies with children with congenital maxillofacial disorders to evaluate if their symmetry is within the normal range.

To our knowledge, no research has been performed regarding the symmetry of the palatal area during growth in infants in their first year. The goal of this study is to investigate whether the symmetry of the palate or alveolar process changes during the first year of life.

## Materials and methods

The study population consisted of 70 infants (36 males) who were derived from an interdisciplinary prospective longitudinal growth and development study at the VU Medical Center in Amsterdam, The Netherlands, by Kramer et al. [9, 10]. The infants were born full term between 1985 and 1988, were Caucasian, and had no craniofacial anomalies and no first- to third-degree relative with an oral cleft. Earlier studies of Kramer and Heidbϋchel et al. already described the characteristics of this study population more extensively [10, 11]. For each infant, plaster casts of the palate were collected at 0, 3, 6, 9, and 12 months after birth.

All data were anonymized prior to analysis. Approval from the regional institutional review board was obtained for this study. This study was conducted in compliance with the World Medical Association Declaration of Helsinki on medical research ethics (2016-2654).

### Data acquisition

All maxillary models were digitized using the 3Shape R500® 3D Dental Laser scanner (3Shape, Copenhagen, Denmark). Scans were made using the high-resolution setting, producing a spatial resolution of 0.01 mm as specified by the manufacturer.

### Palatal segmentation

Before symmetry analysis could be performed, the palatal surface was extracted from the digitized 3D maxillary model. The outline of the palate was determined using a semiautomatic technique which detects the alveolar ridge as described by Bruggink et al. [11]. The alveolar ridge acts as the border of the palate. In summary, this technique uses five landmarks which are manually indicated on the maxilla, namely, two tuberosity points (T), two cuspid points (C), and the most frontal point of the alveolus (A). These points are then used by the algorithm to estimate the most likely location for the alveolar crest. Additionally, a manual landmark was placed on the posterior part of the raphe mediana. The line crossing this raphe landmark and the frontal point will act as an estimation for the raphe mediana, which is used to calculate the medial plane.

**Fig. 1:**
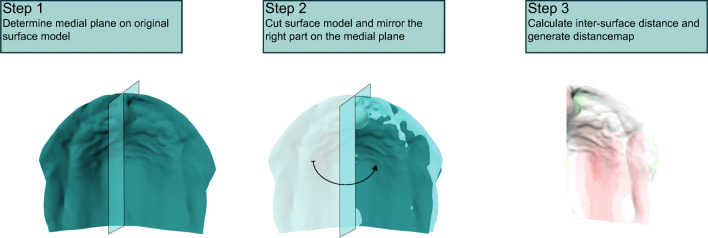
The three main steps to determine the palatal symmetry. 1) The reference frame is calculated with the manual placed landmark. The medial plane is then used to divide the palate in two hemispheres. 2) The right side of the palate is mirrored toward the left side. 3) The distances between the datapoints of the hemispheres are calculated, and a color-coded inter-surface distance map is created

**Fig. 2:**
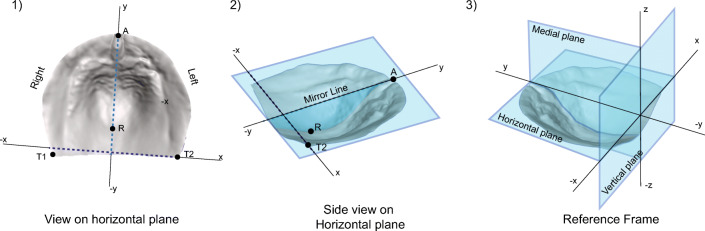
Visualization of the reference frame in three different views. 1) The *x*-axis is located on the line created by both tuberosity points and the *y*-axis between the center of the tuberosity points and the frontal point. 2) The *z*-axis is finally determined by the occlusal plane based on the five placed landmarks. 3) The complete reference frame with all its planes drawn

A symmetry analysis of the maxilla was performed with the use of custom-made software created with MATLAB (MATLAB 2019a, The MathWorks, Inc., Natick, Massachusetts, USA). The analysis consisted of three main steps, which are explained in detail below and illustrated in Figs. 1 and 2.

### Step 1: Creation of a reference frame

In order to mirror the 3D palatal surface, it is important to align it properly in a reference frame to create the midline for the mirroring process. This reference frame is based on three perpendicular planes, the horizontal plane (*z* = 0), the vertical plane (*y* = 0), and the medial plane (*x* = 0). The horizontal plane is determined as the occlusal plane constructed from both tuberosity points and the frontal point. The medial plane, which is used for mirroring, is computed as a plane perpendicular to the horizontal plane and along with the line between the frontal and raphe landmark. The vertical plane is computed as a plane perpendicular to the horizontal and medial plane and crosses the left tuberosity point. The right tuberosity point is not used while determining this plane to maintain a perpendicular frame. This reference frame is illustrated in Fig. 2.

### Step 2: Preparing the palatal mesh

The alveolar crest determined by the algorithm was used to extract the palate from the initial digitized model. Afterward, 3D measurement points were evenly spread over the palatal model with a distance of 0.1 mm between each other, which will be used as starting points for the distance calculations. To create a mirrored palatal model, the original palatal model was duplicated and the medial plane of the reference frame was used to mirror the right hemisphere of the palate to the left side.

### Step 3: Creating the distance map

To quantify the differences, inter-surface distances were calculated between the original and mirrored palatal surface which was performed by creating a line perpendicular to the surface on each measurement point. These lines were extended until they reached the mirrored surface. The length of each line indicates the local distance from the original toward the mirrored surface model. The inter-surface difference was visualized using a color-coded distance map in which red colors indicate a negative difference and green colors a positive difference between the two surfaces. An example of one of the distance maps is shown in Fig. 1, step 3.

Additionally, the palatal area of the original palate was measured. The palatal area was divided in half using the medial plane, resulting in two hemispheres. The areas of both hemispheres were compared and analyzed. Finally, these two hemispheres were used to calculate the anterior and posterior palatal width. The anterior width was measured as the shortest distance between the cuspid landmark and the medial plane. The posterior width was defined as the shortest distance between the tuber landmark and the medial plane. A list of all parameters is shown in Table 1.

**Table 1:** A list of parameters, with their description, indicating symmetry used in this study

Parameter	Description
Average inter-surface distance (mm)	The average distance between each datapoint in the mirrored surface toward the original model of the individual patient
C-C symmetry (mm)	The difference between the distance between the cuspid landmarks and the raphe mediana
T-T symmetry (mm)	The difference between the distance between the tuber landmarks and the raphe mediana
Area symmetry (mm^2^)	The difference between the area between both hemispheres defined by the raphe mediana

### Statistical analysis

To assess the asymmetry between the original and mirrored surface, a Student’s *t* test was performed if the absolute average differed from zero. Additionally, the 95th percentile of each model was calculated and averaged per group to indicate the presence of local abnormalities.

Symmetry differences for all parameters between the five age groups were tested with an ANOVA test in SPSS 25 (IBM Corp. Released 2017. IBM SPSS Statistics for Windows, Version 25.0. Armonk, NY: IBM Corp). Tukey’s post hoc tests were performed to see individual differences between the groups. For all tests, the significance level was set at *p* < 0.05.

### Intra- and interobserver reliability

The reliability was tested by repeating the segmentation and mirroring procedure for 20 randomly chosen maxillary models. This was done by the same observer after a time interval of 2 weeks for the intraobserver difference. A second observer performed the measurements as well for the interobserver difference. Systematic differences were calculated with a paired sample *t* test, and the Dahlberg coefficient was used to investigate the variance.

## Results

From the total of 70 patients, 350 maxillary models were included in this study and grouped in 5 different age categories. Forty-six models were excluded due to loss of follow-up (19), low-quality impressions (17), and segmentation errors, occurring when the algorithm could not accurately determine the alveolar crest (10). Mirroring and analysis succeeded for all the remaining 304 models.

**Fig. 3:**
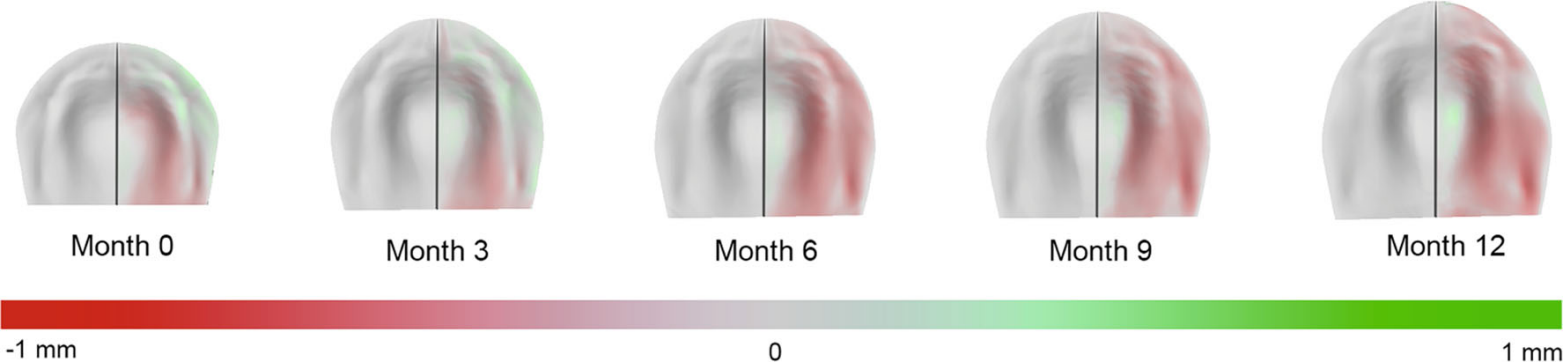
An overview of the average distance maps for 0, 3, 6, 9, and 12 months

In all parameters, small amounts of asymmetry can be observed. As shown in Table 2 and visualized in Fig.3, the mean absolute difference between the original and mirrored surface ranged between 0.23 and 0.30 mm for all age groups. Regarding the 95th percentile, the difference ranged from 0.63 to 0.81 mm. The ANOVA test showed no significant change of the inter-surface distance during the first-year development in infants (*p* = 0.33). Additional post hoc Tukey tests did not show any significant changes. The ANOVA and Tukey test results can be seen in the Appendix section 1 and 2.

**Table 2:** The results of the inter-surface distances for each age group

Parameter	Age in months	*N*	Mean	Standard deviation	Mean absolute deviation	95th percentile of the absolute values	95% CI	*p*
Average inter-surface distance (mm)	0	61	− 0.08	0.32	0.24	0.69	[− 0.14 to 0.02]	0.01*
	3	60	− 0.06	0.24	0.23	0.63	[− 0.11 to 0.00]	0.04*
	6	64	− 0.12	0.27	0.27	0.69	[− 0.18 to − 0.06]	< 0.01*
	9	58	− 0.12	0.29	0.30	0.81	[− 0.21 to − 0.04]	< 0.01*
	12	61	− 0.13	0.27	0.25	0.77	[− 0.18 to − 0.09]	< 0.01*

As seen in Table 3, the analysis of the width of the palate as well as the palatal area measurements indicated that the left palatal hemisphere was more prominent. Both the anterior and posterior width of the left hemisphere were larger compared with the contralateral side. The same could be seen when analyzing the area of the palatal hemisphere. However, the areal and width differences were small and not statistically significant for all age groups.

**Table 3:** The results of the hemispherical symmetry analysis for each age group

Parameter	Age in months	*N*	Mean difference between right and left	Standard deviation	95% CI	*p*
C-C symmetry (mm)	0	61	− 0.15	0.77	[− 0.35 to 0.04]	0.13
	3	60	− 0.05	0.63	[− 0.22 to 0.11]	0.51
	6	64	− 0.23	0.90	[− 0.46 to − 0.01]	0.04*
	9	58	− 0.40	0.85	[− 0.63 to − 0.18]	< 0.01*
	12	61	− 0.23	0.73	[− 0.42 to − 0.01]	0.02*
T-T symmetry (mm)	0	61	− 0.21	0.98	[− 0.46 to 0.05]	0.11
	3	60	− 0.27	1.00	[− 0.53 to 0.01]	0.04*
	6	64	− 0.45	1.34	[− 0.78 to − 0.12]	< 0.01*
	9	58	− 0.27	1.39	[− 0.64 to 0.10]	0.15
	12	61	− 0.66	1.17	[− 0.96 to − 0.36]	< 0.00*
Area symmetry (mm^2^)	0	61	− 4.47	19.26	[− 9.41 to 0.46]	0.08
	3	60	0.17	18.89	[− 4.71 to 5.05]	0.94
	6	64	− 5.48	18.94	[− 10.21 to − 0.75]	0.02*
	9	58	− 8.39	24.85	[− 14.93 to − 1.86]	0.01*
	12	61	−4.94	20.29	[− 10.14 to 0.25]	0.06

The *p* value indicated if the mean differed from 0

The intra- and interobserver reliability analysis showed low absolute mean differences between all measurements, which can be found in Table 4. None of the differences were significant.

**Table 4:** Intra- and interobserver differences between the absolute average inter-surface distance and the 95% interval

Parameter	Mean difference	95% CI	*p*	Dahlberg coefficient
Intraobserver variability				
C-C symmetry (mm)	− 0.23	[− 0.52 to 0.06]	0.12	0.46
T-T symmetry (mm)	0.02	[− 0.40 to 0.40]	0.93	0.62
Area symmetry (mm^2^)	− 0.74	[− 5.29 to 3.80]	0.74	6.72
Average inter-surface distance (mm)	0.03	[− 0.03 to 0.09]	0.35	0.09
Interobserver variability				
C-C symmetry (mm)	− 0.20	[− 0.44 to 0.04]	0.09	0.38
T-T symmetry (mm)	0.08	[− 0.64 to 0.80]	0.82	1.06
Area symmetry (mm^2^)	− 0.29	[− 11.62 to 11.04]	0.96	16.69
Average inter-surface distance (mm)	− 0.05	[− 0.19 to 0.09]	0.43	0.21

## Discussion

The aim of this study was to investigate palatal symmetry in infants and changes of symmetry during growth in the first year of life. Asymmetry was assessed in two steps: by mirroring the palate on its medial plane and comparing the mirrored and original surface and widths and areas. In this population, a small absolute asymmetry ranging from 0.23 to 0.30 mm between the original and mirrored surface was observed. The 95th percentile was used to check for local asymmetries which otherwise would not be visible in the averaged data. This value did not exceed 0.81 mm, and some of this can be explained by the deviations in the palatal rugae where no bilateral symmetry is present [12]. Aside from the surface symmetry, the other measurements showed a marginally larger left hemisphere in comparison with its counterpart but were not all found significant. It can be noted that most significant differences were present at the second half of the year, which could be explained by the fact that environmental factors are having an influence on the symmetry. Other studies are not unanimous about the side of asymmetry. For example, Kent et al. [13] showed a small, not significant, increase of right hemisphere width, which did not exceed 0.7 mm. In an earlier study of Vig et al. [14] who investigated the facial symmetry in cephalograms showed a larger asymmetry on the palatal side, which was in line with the results of our study. The areal differences between the hemispheres were small (within the measurement error); it can be concluded that these differences are not clinically relevant.

Aside from the measurement error, it is thought that fluctuating asymmetry can induce small deviations in the human bilateral symmetry. Fluctuating asymmetry consist of small random deviations from perfect symmetry, whose extend can reflect the developmental stability of the organism. Environmental and genetic stresses could increase this kind of asymmetry, as is the case in cleft palate and Down syndrome [15, 16]. But as this study describes a healthy population with infants, who are only briefly exposed to environmental factors, it could be considered that this influence is minimal.

Using the ANOVA test, no significant differences between each of the groups were found, indicating that the amount of asymmetry is not changing in the first year of life. The small, non-significant increase of the mean difference and 95th percentile in the inter-surface distance can be explained by the normal growth of the infant. When the palate is growing, the inter-surface differences are increasing as well. However, this observation was not seen within the area and width measurements, which could be due to the number of measurements. The inter-surface distance is an average of thousands of measurements, while the other measurements are only singular.

The tool used for the analysis of symmetry required manual landmark identification to extract the palatal area from the 3D model [11]. A previous study showed that manual landmarking introduces limited intra- and interobserver variability within this tool. Determination of the alveolar arch showed no significant intraobserver difference (− 0.50 mm); however, the interobserver difference was found to be significant (2.06 mm). As the confidence intervals were small and the differences were below 4% of the total arch, this was considered to be clinically irrelevant. Furthermore, the intra- and interobserver analysis in that study showed a very strong correlation with a correlation coefficient of 0.91 and 0.96, respectively [11]. This was the only manual intervention which was needed to calculate the inter-surface distances, meaning the placement of the landmarks is the key for a good comparison. For the reproducibility of the distance maps, a small difference in the absolute mean difference was found of 0.03 mm (Dahlberg = 0.09) within observers and − 0.05 mm (Dahlberg = 0.21) between observers. None of the measurements in the reliability analysis were statistically different.

A drawback of the described method is that currently it is unknown in which direction the found asymmetry is present, e.g., is the difference more prominent in the horizontal (width of the maxilla) or vertical direction (height of the maxilla). The measurement for asymmetry of the palate was calculated as a mean of the complete surface. However, it could be possible that asymmetry is only present in a specific area of the palate. Future research could divide the palate in several anatomical areas, for example, based on the embryological development, where each will have its own asymmetry index.

One of the applications of this study could be to evaluate treatment outcome in patients with a unilateral or bilateral cleft treated with nasoalveolar molding (NAM). The goal of NAM is to achieve a more symmetrical position of the maxillary segments and the nose by molding the maxillary segments and nasal cartilage and stretching the columella. The intra-oral molding plates are modified or replaced weekly during treatment to bring the alveolar segments closer to the desired location [17–19]. Fuchigami et al. [17] investigated the restoration of symmetry by using this technique in patients born with unilateral cleft lip and palate (ULCP). They compared the change in landmark locations before and after surgery. Because only landmarks were used to assess the symmetry, a lot of information of the 3D surface remained unused. The techniques described in the present study can provide detailed information about the influence of devices on the symmetry of the maxilla over time. Furthermore, if the influence of the NAM device could be modeled, less dental impressions and possibly fewer patient visits are needed to correct the intra-oral molding plate as the shape shift can be predicted.

With the presented method, the palatal symmetry in healthy infants was assessed. This data gives more insight into the normal maxillary arch and palatal development. The proposed method can be used in future comparative studies with other craniofacial disorders. Furthermore, modeling of these data can help in predicting the growth pattern, which may lead to improved treatment protocols for children with craniofacial anomalies. It should be stated, however, that the treatment goal for congenital anomalies should not be a perfect symmetry as this is also not the case in healthy infants. The focus of the treatment should still be to optimize functional impairments and achieve esthetically acceptable results.

## Conclusion

In this population of infants, only a small degree of palatal asymmetry was found which can be considered normal and clinically irrelevant. The results of this study can serve as reference data for comparative studies to investigate palatal differences in craniofacial abnormalities.

## Electronic supplementary material

ESM 1 (DOCX 31 kb)
