# Supplementary material for: Symmetry of palatal shape during the first year of life in healthy infants
Source: Clin Oral Investig. 2020 Jun 24;25(3):1069–76. doi: 10.1007/s00784-020-03403-4 (PMC7878251; doi:10.1007/s00784-020-03403-4)
Supplement: Supplementary file 1 — (DOCX 31 kb) [file 784_2020_3403_MOESM1_ESM.docx]

**Appendix**

**Symmetry of Palatal Shape During the First Year of Life in Healthy Newborns**

**R. Bruggink** ^1,2*^,

**F. Baan** ^1,2^,

**G.J.C. Kramer** ^3^,

**A.M. Kuijpers-Jagtman** ^6,7,8^,

**S.J. Bergé** ^4,5^,

**T.J.J. Maal** ^2,4^,

**E.M. Ongkosuwito** ^1,5^

# Appendix 1, Results of the ANOVA tests

| **ANOVA** | | | | | | |
| --- | --- | --- | --- | --- | --- | --- |
|  | | Sum of Squares | df | Mean Square | F | Sig. |
| Mean inter-surface distance (Total) | Between Groups | .559 | 4 | .140 | 1.800 | .129 |
|  | Within Groups | 23.206 | 299 | .078 |  |  |
|  | Total | 23.765 | 303 |  |  |  |
| Symmetry at C-C (Right - Left) | Between Groups | 4.127 | 4 | 1.032 | 1.704 | .149 |
|  | Within Groups | 181.086 | 299 | .606 |  |  |
|  | Total | 185.213 | 303 |  |  |  |
| Symmetry at T-T (Right - Left) | Between Groups | 9.235 | 4 | 2.309 | 1.607 | .172 |
|  | Within Groups | 429.582 | 299 | 1.437 |  |  |
|  | Total | 438.817 | 303 |  |  |  |
| Area symmetry (Right - Left) | Between Groups | 2058.034 | 4 | 514.508 | 1.146 | .335 |
|  | Within Groups | 134216.533 | 299 | 448.885 |  |  |
|  | Total | 136274.566 | 303 |  |  |  |

# Appendix 2, Results of the post-hoc Tukey HSD

| **Multiple Comparisons** | | | | | | | |
| --- | --- | --- | --- | --- | --- | --- | --- |
| Tukey HSD | | | | | | | |
| Dependent Variable | (I) nMonth | (J) nMonth | Mean Difference (I-J) | Std. Error | Sig. | 95% Confidence Interval | |
|  |  |  |  |  |  | Lower Bound | Upper Bound |
| Mean inter-surface distance (Total) | 0 | 3 | -.02666 | .04329 | .973 | -.1455 | .0922 |
|  |  | 6 | .03543 | .04260 | .921 | -.0815 | .1524 |
|  |  | 9 | .04182 | .04367 | .874 | -.0780 | .1617 |
|  |  | 12 | .05263 | .04311 | .739 | -.0657 | .1710 |
|  | 3 | 0 | .02666 | .04329 | .973 | -.0922 | .1455 |
|  |  | 6 | .06209 | .04279 | .595 | -.0553 | .1795 |
|  |  | 9 | .06848 | .04384 | .523 | -.0518 | .1888 |
|  |  | 12 | .07929 | .04329 | .357 | -.0395 | .1981 |
|  | 6 | 0 | -.03543 | .04260 | .921 | -.1524 | .0815 |
|  |  | 3 | -.06209 | .04279 | .595 | -.1795 | .0553 |
|  |  | 9 | .00639 | .04317 | 1.000 | -.1121 | .1249 |
|  |  | 12 | .01720 | .04260 | .994 | -.0997 | .1341 |
|  | 9 | 0 | -.04182 | .04367 | .874 | -.1617 | .0780 |
|  |  | 3 | -.06848 | .04384 | .523 | -.1888 | .0518 |
|  |  | 6 | -.00639 | .04317 | 1.000 | -.1249 | .1121 |
|  |  | 12 | .01081 | .04367 | .999 | -.1090 | .1307 |
|  | 12 | 0 | -.05263 | .04311 | .739 | -.1710 | .0657 |
|  |  | 3 | -.07929 | .04329 | .357 | -.1981 | .0395 |
|  |  | 6 | -.01720 | .04260 | .994 | -.1341 | .0997 |
|  |  | 9 | -.01081 | .04367 | .999 | -.1307 | .1090 |
| Symmetry at C-C (Right - Left) | 0 | 3 | -.09716 | .14216 | .960 | -.4873 | .2930 |
|  |  | 6 | .08150 | .13990 | .978 | -.3025 | .4655 |
|  |  | 9 | .25104 | .14339 | .405 | -.1425 | .6446 |
|  |  | 12 | .08023 | .14157 | .980 | -.3083 | .4688 |
|  | 3 | 0 | .09716 | .14216 | .960 | -.2930 | .4873 |
|  |  | 6 | .17866 | .14050 | .709 | -.2069 | .5643 |
|  |  | 9 | .34821 | .14397 | .113 | -.0469 | .7433 |
|  |  | 12 | .17739 | .14216 | .723 | -.2128 | .5675 |
|  | 6 | 0 | -.08150 | .13990 | .978 | -.4655 | .3025 |
|  |  | 3 | -.17866 | .14050 | .709 | -.5643 | .2069 |
|  |  | 9 | .16955 | .14174 | .754 | -.2195 | .5586 |
|  |  | 12 | -.00127 | .13990 | 1.000 | -.3852 | .3827 |
|  | 9 | 0 | -.25104 | .14339 | .405 | -.6446 | .1425 |
|  |  | 3 | -.34821 | .14397 | .113 | -.7433 | .0469 |
|  |  | 6 | -.16955 | .14174 | .754 | -.5586 | .2195 |
|  |  | 12 | -.17081 | .14339 | .756 | -.5643 | .2227 |
|  | 12 | 0 | -.08023 | .14157 | .980 | -.4688 | .3083 |
|  |  | 3 | -.17739 | .14216 | .723 | -.5675 | .2128 |
|  |  | 6 | .00127 | .13990 | 1.000 | -.3827 | .3852 |
|  |  | 9 | .17081 | .14339 | .756 | -.2227 | .5643 |
| Symmetry at T-T (Right - Left) | 0 | 3 | .06770 | .21615 | .998 | -.5255 | .6609 |
|  |  | 6 | .24339 | .21272 | .783 | -.3404 | .8272 |
|  |  | 9 | .06315 | .21802 | .998 | -.5352 | .6615 |
|  |  | 12 | .45888 | .21525 | .209 | -.1319 | 1.0496 |
|  | 3 | 0 | -.06770 | .21615 | .998 | -.6609 | .5255 |
|  |  | 6 | .17568 | .21362 | .924 | -.4106 | .7620 |
|  |  | 9 | -.00456 | .21890 | 1.000 | -.6053 | .5962 |
|  |  | 12 | .39117 | .21615 | .370 | -.2020 | .9844 |
|  | 6 | 0 | -.24339 | .21272 | .783 | -.8272 | .3404 |
|  |  | 3 | -.17568 | .21362 | .924 | -.7620 | .4106 |
|  |  | 9 | -.18024 | .21551 | .919 | -.7717 | .4112 |
|  |  | 12 | .21549 | .21272 | .849 | -.3683 | .7993 |
|  | 9 | 0 | -.06315 | .21802 | .998 | -.6615 | .5352 |
|  |  | 3 | .00456 | .21890 | 1.000 | -.5962 | .6053 |
|  |  | 6 | .18024 | .21551 | .919 | -.4112 | .7717 |
|  |  | 12 | .39573 | .21802 | .367 | -.2026 | .9941 |
|  | 12 | 0 | -.45888 | .21525 | .209 | -1.0496 | .1319 |
|  |  | 3 | -.39117 | .21615 | .370 | -.9844 | .2020 |
|  |  | 6 | -.21549 | .21272 | .849 | -.7993 | .3683 |
|  |  | 9 | -.39573 | .21802 | .367 | -.9941 | .2026 |
| Area symmetry (Right - Left) | 0 | 3 | -4.64451 | 3.72948 | .725 | -14.8801 | 5.5910 |
|  |  | 6 | 1.01058 | 3.67026 | .999 | -9.0624 | 11.0836 |
|  |  | 9 | 3.92133 | 3.76176 | .835 | -6.4028 | 14.2455 |
|  |  | 12 | .47341 | 3.71404 | 1.000 | -9.7198 | 10.6666 |
|  | 3 | 0 | 4.64451 | 3.72948 | .725 | -5.5910 | 14.8801 |
|  |  | 6 | 5.65508 | 3.68588 | .541 | -4.4608 | 15.7710 |
|  |  | 9 | 8.56584 | 3.77701 | .158 | -1.8001 | 18.9318 |
|  |  | 12 | 5.11792 | 3.72948 | .646 | -5.1176 | 15.3535 |
|  | 6 | 0 | -1.01058 | 3.67026 | .999 | -11.0836 | 9.0624 |
|  |  | 3 | -5.65508 | 3.68588 | .541 | -15.7710 | 4.4608 |
|  |  | 9 | 2.91076 | 3.71854 | .935 | -7.2948 | 13.1163 |
|  |  | 12 | -.53716 | 3.67026 | 1.000 | -10.6102 | 9.5359 |
|  | 9 | 0 | -3.92133 | 3.76176 | .835 | -14.2455 | 6.4028 |
|  |  | 3 | -8.56584 | 3.77701 | .158 | -18.9318 | 1.8001 |
|  |  | 6 | -2.91076 | 3.71854 | .935 | -13.1163 | 7.2948 |
|  |  | 12 | -3.44792 | 3.76176 | .890 | -13.7721 | 6.8762 |
|  | 12 | 0 | -.47341 | 3.71404 | 1.000 | -10.6666 | 9.7198 |
|  |  | 3 | -5.11792 | 3.72948 | .646 | -15.3535 | 5.1176 |
|  |  | 6 | .53716 | 3.67026 | 1.000 | -9.5359 | 10.6102 |
|  |  | 9 | 3.44792 | 3.76176 | .890 | -6.8762 | 13.7721 |
